# Psychiatrists' Attitudes Toward Disruptive New Technologies: Mixed-Methods Study

**DOI:** 10.2196/10240

**Published:** 2018-12-14

**Authors:** Alexis Bourla, Florian Ferreri, Laetitia Ogorzelec, Charles-Siegfried Peretti, Christian Guinchard, Stephane Mouchabac

**Affiliations:** 1 Department of Adult Psychiatry and Medical Psychology Sorbonne Université Saint-Antoine Hospital, Assistance Publique Hôpitaux de Paris Paris France; 2 Sociology and Anthropology Laboratory University of Burgundy Franche-Comté Besançon France

**Keywords:** acceptability, clinical decision support systems, computerized adaptive testing, digital phenotype, ecological momentary assessment, machine learning, mobile phone, professional culture

## Abstract

**Background:**

Recent discoveries in the fields of machine learning (ML), Ecological Momentary Assessment (EMA), computerized adaptive testing (CAT), digital phenotype, imaging, and biomarkers have brought about a new paradigm shift in medicine.

**Objective:**

The aim of this study was to explore psychiatrists’ perspectives on this paradigm through the prism of new clinical decision support systems (CDSSs). Our primary objective was to assess the acceptability of these new technologies. Our secondary objective was to characterize the factors affecting their acceptability.

**Methods:**

A sample of psychiatrists was recruited through a mailing list. Respondents completed a Web-based survey. A quantitative study with an original form of assessment involving the screenplay method was implemented involving 3 scenarios, each featuring 1 of the 3 support systems, namely, EMA and CAT, biosensors comprising a connected wristband-based digital phenotype, and an ML-based blood test or magnetic resonance imaging (MRI). We investigated 4 acceptability domains based on International Organization for Standardization and Nielsen models (usefulness, usability, reliability, and risk).

**Results:**

We recorded 515 observations. Regarding our primary objective, overall acceptability was moderate. MRI coupled with ML was considered to be the most useful system, and the connected wristband was considered the least. All the systems were described as risky (410/515, 79.6%). Regarding our secondary objective, acceptability was strongly influenced by socioepidemiological variables (professional culture), such as gender, age, and theoretical approach.

**Conclusions:**

This is the first study to assess psychiatrists’ views on new CDSSs. Data revealed moderate acceptability, but our analysis shows that this is more the result of the lack of knowledge about these new technologies rather than a strong rejection. Furthermore, we found strong correspondences between acceptability profiles and professional culture profiles. Many medical, forensics, and ethical issues were raised, including therapeutic relationship, data security, data storage, and privacy risk. It is essential for psychiatrists to receive training and become involved in the development of new technologies.

## Introduction

Recent discoveries in the fields of genetics, imaging, and biomarkers, together with the development of medical informatics, are leading us to rethink psychiatry. The practices, representations, ethics, and beliefs of practitioners could be disrupted. In science, the ability to predict [1] the occurrence of a morbid event opens up important perspectives—not only preventive or curative but also ethical. At the interface between electronic health, new technologies, and clinical observation, a large number of new tools are currently being developed for the early detection of psychotic or mood disorders and for the prediction of their course. A growing number of studies are reporting on the use of computerized assistance, especially artificial intelligence, in the form of new clinical decision support systems (CDSSs), and current changes to these systems tend to associate 2 concepts: digital phenotyping and machine learning (ML).

Torous and Gualtieri underlined the potential usefulness of connected objects in the field of mental health [2], as many devices now include multiple sensors (accelerometer, heart rate sensor, sleep tracker, skin conductance sensor, light sensor, etc). The prospect of being able to gather real-time physiological data from fitness trackers as well as from symptom checkers in smartwatches is an attractive one, and there is increasing interest in using real-time patient data as biomarkers for illness. Their team recently developed the concept of the digital phenotype of pathology. This refers to capture by computerized measurement tools of specific characteristics of psychiatric disorders [3,4]. Some behaviors or symptoms may be objectifiable and quantifiable by computer tools, thereby constituting an e-semiotic (semiotics mediated by computerized tools). Thus, the graphorrhea observed in manic episodes can be reflected in an increase in the number of short service message text messages sent, and depressive psychomotor retardation can be assessed by an accelerometer [5].

Sensor miniaturization and the ubiquitous use of smartphones mean that it is now possible to collect a large amount of data that psychiatrists had never previously been able to access. Models based on these new signs are emerging in the field of schizophrenia [6] and mood disorders [5]. These passive data are collected in background tasks for which no intervention is necessary. To reduce observer bias, the individual is not always aware when the data are being collected. Detection may involve a mobile phone and its onboard sensors (global positioning system, accelerometer, verbal flow detector, etc) or connected wearable objects that allow biometric monitoring to take place in the real-time. Data can also be collected actively by Ecological Momentary Assessment (EMA) on a smartphone, but the collection of live data requires action on the part of the patient. EMA involves the evaluation of symptoms from day to day in the patient’s habitual environment, and as they evaluate themselves (right then, not later; right there, not elsewhere) there can be no recall biases [7,8]. This method allows a much more individualized approach to introducing precision diagnosis in psychiatry [9], as symptoms are connected through a system of causal relations, with symptoms impacting on each other (ie, insomnia impacting on depressive symptoms, depressive symptoms impacting on anxiety symptoms, hallucinations impacting on delusions, etc). In addition, many symptoms may be context-dependent (ie, increasing alcohol craving when approaching a bar). EMA can capture dimensional variation in mental states in response to other mental states or environmental variation, resulting in a diagnosis that is both precise and contextual.

All these data, far too copious to be analyzed manually, can be processed by computer software, allowing patients to be classified according to their illness. As we have seen, new technologies (smartphone, computers, and biomarkers) and the parallel expansion of medical informatics and artificial intelligence have brought about a paradigm shift toward a more personalized and predictive form of medicine [10]. But if some disorders can be recognized by computer models and if diseases or relapses can be detected earlier or more precisely by machines or smartphones, what role will health care providers play in the future?

The advent of these technologies calls into question psychiatrists’ professional culture. This sociological concept, derived from the sociology of professions, refers to the fact that professionals refer not only to theoretical knowledge or experience but also to a set of customs, a specific language, and a set of common values [11,12]. According to sociology, a professional activity profoundly influences the identity of the individuals who exercise it. These individuals are defined by their membership of the profession, conceived of as a fully-fledged social group and a culture bearer, sharing values and beliefs as well as a common way of expressing them [13,14]. In that aspect, some authors are already suggesting that psychiatrists are an endangered species [15]. Indeed, psychiatrists diverge from other medical specialties in terms of the predominance of clinical reasoning, the lack of specific or valid imaging techniques or biological tests, and the importance given to intuition, clinical sensitivity, and the therapeutic relationship. From this point of view, the psychotherapeutic dimension of the psychiatric interview could be challenged by these new technologies.

To our knowledge, there has been little research in this area, and although several studies have recently focused on the acceptability of these technologies for patients or patient compliance, potential prescribers have never been questioned on the subject. The acceptability of these technologies must, therefore, be assessed at different levels, namely, usability (intention to use), utility (technology’s contribution), reliability (including accuracy, effectiveness, and efficiency), and risk, which constitute important dimensions of medical reasoning.

The main objective of this study was to analyze psychiatrists’ perspectives on these new technologies by assessing the acceptability of 3 CDSSs: (1) smartphone-based EMA, (2) connected wristband-based digital phenotype, and (3) ML-based prediction magnetic resonance imaging (MRI) or blood test. We used a model specifically developed for this purpose with a pluridisciplinary approach (psychiatric and sociological). The secondary objective was to characterize the factors affecting this acceptability and, consequently, indirectly affecting the psychiatrists’ professional culture.

## Methods

### Study Design

We conducted a qualitative and quantitative study via a computerized survey (Google-Form), in collaboration with the Sociology and Anthropology Laboratory of the University of Burgundy Franche-Comté (LaSA, UBFC).

### Target Population and Sample Composition

This study focused on a population of psychiatrists working in France. They ranged from residents to senior psychiatrists, working in psychiatric facilities, general or university hospitals, or private practices. Requirement of Ethical Committee’s approval was waived.

### Survey Development

We used an original form of assessment, with 2 researchers at Sociology and Anthropology Laboratory of the University of Burgundy Franche-Comté, based on the screenplay method, an assessment method used to expose respondents to challenging and problematic clinical cases, in order to ask them to express what should be done or what they themselves have done to act with competency in such situations. By confronting the psychiatrists with systems or devices that are still essentially restricted to the field of research, we were able to review some aspects of reality that are not captured by other types of evocation. The 3 scenarios used here allow practitioners to think about devices that are currently in the research domain and are not used (or little used) in daily routine.

The screenplay method featured 3 clinical case presentations involving new technologies (Table 1 and Multimedia Appendix 1).

All the questions were designed during 3 focus groups including psychiatrists and sociologists and were tested with cross-validation on a sample of psychiatrists working at Saint-Antoine Hospital in Paris, France. The first part of the survey (15 questions) collected epidemiological data: sex, job, place of practice, theoretical and practical training (neurobiological, psychoanalytic, integrative, cognitive behavioral therapy, etc), workplace, year of graduation, and practice area (adult psychiatry, child psychiatry, forensic psychiatry, etc). The second part assessed the acceptability of the support systems and the psychiatrists’ professional culture, with 15 questions per scenario (total of 60 questions). To avoid responder focus being too much on confounding factors (ie, the shortage of health personnel aspect), we asked direct questions about the devices in our questionnaire (Textbox 1). A blank field allowed us to collect qualitative data in the form of feedback at the end of the survey.

### Assessment of Acceptability

The various technologies described above can be studied from a sociological perspective by examining the factors that prevent or, conversely, encourage their use**.** Several dimensions that can influence acceptability were included in an acceptability model specifically developed for the study and inspired by research on human-machine interaction and management information systems, combining the Nielsen, International Organization for Standardization, and Shackel models [21,22] (see Multimedia Appendix 2). The variables most frequently associated with acceptability are usability (ie, intention to use), supposed usefulness, and reliability [23-25]. In our model, we assessed 4 variables: usefulness, usability, reliability, and risk (Textbox 1). For each variable, participants responded to the questions on a Likert-like scale ranging from 1 to 6, depending on the item. To gauge the acceptability of each system, we calculated a composite score with 3 values (positive, intermediate, and negative).

### Psychiatrists’ Professional Culture

The purpose of each item was to bring out the characteristics of the sociological concept known as psychiatrists’ professional culture: what made them psychiatrists, with which technologies they would refuse to compromise, and how they saw themselves in relation to other specialists. We took 2 major areas of professional culture into account. First, we investigated the psychiatrists’ scientificity level, reflected by the use of biometric data, MRI, blood tests, and physical examinations (assuming that the more scientifically-minded the psychiatrists are, the more willing they are to use complementary examinations). Second, we probed the psychiatrists’ specific relationship with technology by analyzing the hopes and fears generated by these new tools (ie, did they think that these technologies would help them or replace them?; see Textbox 2).

**Table 1:** Screenplay method.

Scenario	Objective
1. Detection and diagnosis of a mood disorder using computerized adaptive testing [16] and smartphone-based Ecological Momentary Assessment in a young patient suspected of having a depressive disorder [17]	To evaluate the acceptability of a machine instead of a psychiatrist for making a diagnosis
2. Early detection of depressive relapse using an electronic connected wristband (biosensors) [18] to assess the digital phenotype of a patient with a recurrent depressive disorder in remission	To investigate the psychiatrists’ views on the intrusion of a connected object between them and their patients. Investigate their views on the device’s ability in early detection
3. Prediction of transition to psychosis in individuals in an at-risk mental state using a machine learning algorithm applied to magnetic resonance imaging [19] or blood test [20]	To evaluate the acceptability of a machine learning-based prediction of disease transition

**Acceptability assessment.**

Scenario 1 (Ecological Momentary Assessment and computerized adaptive testing)

1. In absolute terms, do you think that the devices presented in this scenario (smartphone-based Ecological Momentary Assessment) are useful? Same question for computerized adaptive testing
2. Would you use this type of device for depressive disorder diagnosis?
3. Do you think that the devices presented in this scenario are reliable?
4. Do you think that the devices presented in this scenario are at risk?
5. If yes, why?
6. Do you think that the devices presented in this scenario allow you to do:
  - tasks that waste your time?
  - tasks that you don’t like to do?
  - tasks that you don’t know how to do?

Scenario 2 (Digital phenotype)

1. In absolute terms, do you think that the devices presented in this scenario (connected wristband allowing biometric data collection) are useful?
2. Would you prescribe this type of device for early detection of a depressive relapse?
3. Do you think that the devices presented in this scenario are reliable?
4. Do you think that the devices presented in this scenario are at risk?
5. If yes, why?
6. Do you think that the devices presented in this scenario allow you to do:
  - tasks that waste your time?
  - tasks that you don’t like to do?
  - tasks that you don’t know how to do?

Scenario 3 (Machine learning)

1. In absolute terms, do you think that the devices presented in this scenario (magentic resonance imaging and machine learning) are useful? Same question for blood test
2. Would you prescribe this type of device for psychotic transition prediction?
3. Do you think that the devices presented in this scenario are reliable?
4. Do you think that the devices presented in this scenario are at risk?
5. If yes, why?
6. Do you think that the devices presented in this scenario allow you to do:
  - tasks that waste your time?
  - tasks that you don’t like to do?
  - tasks that you don’t know how to do?

**Professional culture assessment.**

1. For all the 3 scenarios:
  - Do you think that these devices make you lose part of your role?
  - Do you think that these devices could be useful for general practitioners?
  - Do you think these devices influence the therapeutic relationship?
  - Do you think these devices are better than the psychiatrist in that specific matter?
  - Do you think that there is a risk that these devices replace the psychiatrist in that specific matter?
2. Specific for scenario 2:
  - Do you think that this device constitutes an intrusion on the patient’s life?
  - Do you use that kind of data (psychomotor retardation, heart rate, biometrics, etc) in the follow-up of your depressive patients?
3. Specific for scenario 3:
  - Do you think that this kind of probabilistic reasoning based on an algorithm can have a place in your practice?
  - Do you think that the use of this kind of device needs specific technical abilities?

### Data Collection

The survey was created with Google Forms and sent by email to the relevant professionals via several mailing lists (residents’ association, private practice associations, clinical facilities, personal social networks, etc). Respondents could answer via an internet browser. After a short introductory text, the scenarios appeared one after the other, each followed by the corresponding questions. The survey was anonymous and took about 10-15 minutes to complete.

### Data Analysis

We performed an initial descriptive analysis of the population using multiple regression analysis. Comparisons of proportions were carried out using a *z* test with Bonferroni correction. Pearson correlation coefficients were used to analyze correlations between variables. The variables were compared with nonparametric chi-square tests or with Fisher’s test when the conditions for chi-square application were not met, using Microsoft Excel, SPSS v24, and R statistical software. The significance level was set at 5%, such that differences with a *P* value <.05 were deemed to be significant. In order to achieve 95% statistical power with an alpha risk of .05, the bibliographic analysis indicated that 374 participants were required (bearing in mind that there were 12,591 psychiatrists in France in 2016) [26]. Qualitative variables were partially analyzed by LaSA using Modalisa (in press).

## Results

### Survey Implementation

The Web-based survey was available between June 30 and August 8, 2016. A total of 528 responses were received. We excluded 5 empty surveys, 5 duplicates, and 3 incomplete surveys (no responses to at least 1 whole scenario) such that 515 surveys were included in the analysis.

### Demographics

The study population was predominantly female (299/515, 58.1%), mainly composed of young psychiatrists who had already graduated or were set to do so between 2010 and 2020 (342/515, 66.4%), and the majority of practitioners worked in adult psychiatry (270/515, 52.4%). Residents made up a large proportion of the sample (241/515, 46.8%), followed by hospital practitioners (148/515, 28.7%) and private practitioners (49/515, 9.5%). The 2 most common theoretical approaches were “several approaches focusing on neurobiology or cognitive behavioral therapy” and “integrative practice” (see Multimedia Appendix 3).

### Primary Outcome: Acceptability of Support Systems

#### Quantitative Analysis

**Figure 1:**
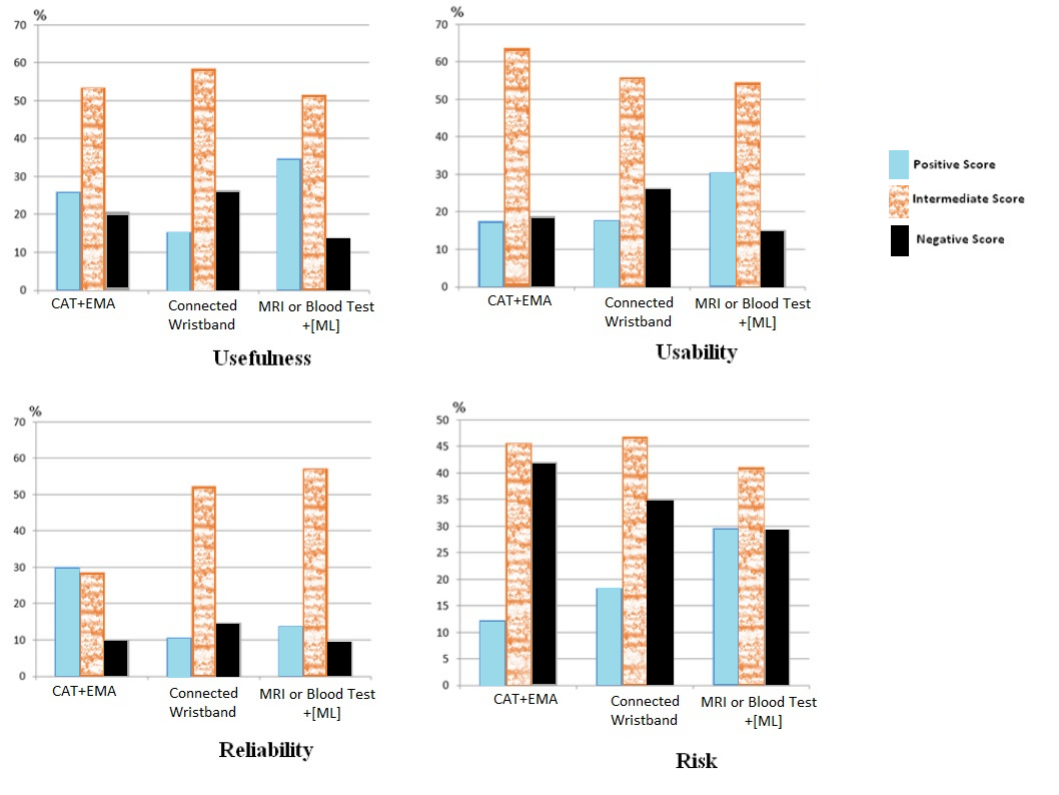
Acceptability and score classification of the 3 scenarios. CAT: computerized adaptive testing; EMA: Ecological Momentary Assessment; ML: machine learning; MRI: magnetic resonance imaging.

The overall acceptability was moderate (Table 2). Positive scores only outweighed negative scores for ML. They did not differ significantly for computerized adaptive testing (CAT) or EMA, and the fewest positive scores were for the connected wristband (Figure 1 and Table 2). MRI coupled with ML was considered to be the most useful system, although when asked about reliability, participants gave CAT most positive scores. All the systems were deemed to be potentially risky (211/515, 41.1%) or risky (198/515, 38.5%). MRI and blood tests had the most favorable risk profile (ie, fewest negative scores). For those who responded that there was a risk (potential or real), the main risks were medical (regardless of the technology), then ethical (especially regarding MRI and blood tests), and finally legal (mainly with regard to the connected wristband).

#### Qualitative Analysis

Qualitative analysis explored the obstacles to the acceptability of these new technologies. There were 3 major issues emerging from the analysis, with a variable distribution according to different scenarios: medical, ethical, and forensic (Textbox 3).

#### Subgroup Analysis

A cross-analysis of the epidemiological data and acceptability profiles was performed (Table 3).

### Secondary Outcome: Characterization of Psychiatrists’ Professional Culture

We asked 3 questions that allowed us to study the place of psychiatrists compared with other medical specialists in terms of scientificity by probing the importance they gave to physical signs (ie, biometric data), technology, and algorithmic thinking. These questions made it possible to distinguish between psychiatrists who:

1. make extensive use of biometric data in their practice,
2. give a prominent place to predictability based on algorithms,
3. view technology not as replacing them but as playing a complementary role.

They also allowed us to highlight the degree of opposition to these dimensions, including affirming the primacy of the human relationship and the refusal or rejection of technology. We were able to place psychiatrists on a continuum running from a medical to psychological subtype and conducted an analysis in which these profiles were crossed with the epidemiological data. This analysis revealed a perfect match between acceptability scores and psychiatrists’ professional culture: the medical profile had the highest acceptability score.

**Table 2:** Acceptability score.

Acceptability domains and technology		Positive score (5-6), %	Intermediate score (3-4), %	Negative score (1-2), %
**Utility**					
	EMA^a^ or CAT^b^	25.9	53.4	20.7
	Connected wristband	15.3	58.4	26.3
	Machine learning	34.7	51.55	13.75
**Usability**					
	EMA or CAT	17.65	63.7	18.8
	Connected wristband	17.7	55.9	26.4
	Machine learning	30.45	54.5	15.05
**Reliability**					
	EMA or CAT	30.05	28.6	9.95
	Connected wristband	10.7	52.3	14.8
	Machine learning	13.75	57.15	9.45
**Risk^c^**					
	EMA or CAT	12.3	45.7	42
	Connected wristband	18.3	46.8	34.9
	Machine learning	29.6	41.1	29.3
**Average**					
	EMA or CAT	21.47	47.85	22.86
	Connected wristband	15.5	53.35	25.6
	Machine learning	27.12	51.07	16.88

^a^EMA: Ecological Momentary Assessment.

^b^CAT: computerized adaptive testing.

^c^For the risk domain, positive and negative scores were inverted.

**Major issues raised by psychiatrists.**

1. Medical:
  - Alteration of the therapeutic relationship
  - Generating anxious counter-reactions (wearable device, prediction)
  - False-positive, false-negative
2. Ethical:
  - Impact of preemptive antipsychotic treatment
  - Impact of predicting a potentially incurable disease
  - Stigmatization risk
  - Used to compensate for increasing shortages of health professionals in some areas
  - Associated in people’s minds with the electronic ankle tagging of prisoners
  - Feeling of being controlled
3. Forensic:
  - Delegate a monitoring task to a machine
  - Data privacy
  - Medical liability

**Table 3:** Cross-analysis of the epidemiological data and acceptability profiles.

Acceptability	Positive scores	Intermediate scores	Negative scores
Sex	Male	N/A^a^	Female
Theoretical approach	Neurobiological	Cognitive behavioral therapy Integrative Systemic	Psychoanalytic
Practice	Adult psychiatry Addiction medicine Geriatric psychiatry	N/A	Child psychiatry Forensic psychiatry
Role	Professor Assistant professor	Hospital practitioner Assistant practitioner Private practitioner	Resident
Year of graduation	1990-2009	2010-2015	2016-2020

^a^N/A: not applicable.

## Discussion

### Principal Findings

We achieved our primary objective of determining the acceptability of new technologies by psychiatrists, and to our knowledge, this is the first study to assess psychiatrists’ views on computerized CDSSs. CDSSs clearly had different acceptability profiles. The connected wristband seemed to have the lowest acceptability profile; CAT and EMA smartphone- based assessment were rated as the most reliable but with the least usability; and ML-based MRI or blood tests were rated as having the greatest usefulness and usability but with the greatest risk. Furthermore, approximately half of the psychiatrists claimed to wait for (and expected to receive) more scientifically validated arguments before reaching any conclusions. This could be a valid argument as the wait-and-see position could be underlying a healthy skepticism. In our opinion, what appears above all in this matter is the lack of knowledge of these techniques among practitioners. Many scientific studies clearly demonstrate the interest in these devices. A recent review of the literature by Faurholt-Jepsen et al [27] regarding the comparative validity of electronic self-assessment techniques over scale or classical clinic assessment found no significant differences between all evaluation methods (smartphone, computer software, internet, and standard clinical scales). According to a review conducted by a team from the Black Dog Institute between 2008 and 2013 [28], reliability and patient acceptability of smartphone-based symptom monitoring is high, with good agreement between electronic measurements and standard diagnostic scales. Similarly, Torous et al [17] demonstrated a good agreement between paper and smartphone versions of the Patient Health Questionnaire, 9^th^ Revision, Beck Depression Inventory, and Quick Inventory of Depressive Symptomatology, with a preference for the smartphone media. Regarding the digital phenotype, the capture of passive data concerns objective criteria of depression recognized by all practitioners (ie, psychomotor retardation in depressive disorder, manic graphorrhea, etc), so it is more a doubt cast on the ability of these devices to “capture” these elements than a rejection of the principle behind it. However, again, several studies scientifically show the validity of this type of data collection. It should also be noted that all the scripts had bibliographic references at the end, allowing responders to learn about these studies.

The subgroup analysis showed that acceptability was strongly mediated by a psychiatrist’s profile and, more specifically, by sex, theoretical approach, mode of practice, role, and experience. One may wonder why the youngest (residents) have a more negative acceptability profile than the others, while intuitively we could think that they would be more favorable to new technologies given their different usage profile. The qualitative sociological analysis of the data shows that it could be a counter-reaction in relation to the feeling of being dispossessed of a recently hard-won knowledge (ie, “If a machine is able to do better than me, then why did I spend 4 years in training?”). This effect diminishes with age.

The questions probing the psychiatrists’ professional culture indicated that a certain type of professional culture corresponded to a certain acceptability profile. Our analysis suggests that there were several subgroups, which differed in almost every aspect, with psychiatrists whose professional culture could be defined as medical at one extreme and those who had a more psychodynamic culture at the other extreme. The question of prediction [29] seems more sensitive for certain categories, especially child psychiatrists and forensics practitioners who are the most reluctant in this area, which illustrates the important current debate on neuroprediction [30,31].

The number of usable responses we collected (N=515) allowed us to have a representative sample of a good size and a geographical distribution that did not influence the data (no significant differences between the regions). Compared with other survey-based studies, this was a large sample as most studies on psychiatrists collect between 50 and 150 responses [32]. The questions were developed in collaboration with sociologists to ensure the relevance of the data we collected and allow for the construction of a sociological hypothesis. Our analysis allowed us to identify a typology of French psychiatrists that featured 2 contrasting schools of thought. The use of quantitative data, including ratings on 3- or 6-point scales, allowed us to undertake a relatively fine-tuned analysis. Furthermore, the questions were developed from a model specifically adapted to medical technology acceptance and inspired by several valid theoretical models [21,22].

Qualitative analysis allows us to explore the different fields of acceptability, raising several constraints. The main obstacle was the psychiatrists’ fear that they would do more harm than good either by generating anxious counter-reactions (especially with regard to the EMA smartphone app and connected wristband) or by creating a risk of overtreatment by diagnosing problems that did not exist. This evokes the notion of self-fulfilling prophecies developed by Robert K Merton [33], which consists, from a false starting hypothesis, of provoking a behavior that makes this initially false hypothesis become true. An anxious reaction may be one of the consequences, which may increase the risk of recurrence, but we could argue that this could also apply to any form of care (including medication). Much of the feedback focused on the third scenario (which had the highest acceptability profile), with questions about which course of action to pursue if the MRI or the blood tests predicted a transition to psychosis and pointing out the risk of jumping to diagnostic conclusions. Several respondents indicated that they would refuse to introduce a preemptive antipsychotic treatment based on a prediction made in this way. Several commented that there was no point in predicting an incurable disease. In fact, this is an interesting result itself, as it shows that the vast majority of psychiatrists interviewed have no idea what to do if there is a risk of psychotic transition. Recent works highlight the importance of a number of measures to limit this risk (fatty acids, low-doses of atypical antipsychotic, active surveillance, open dialogue, etc), and clearly, many psychiatrists are not aware of this [34]. Feedback on the second scenario raised the same questions, with the idea that it is not ethically acceptable for a psychiatrist to delegate a monitoring task to a machine. This technology elicited particular ethical and political considerations based on the notion that these connected wristbands are associated in people’s minds with the electronic ankle tagging of prisoners and that they are part of a political agenda used to compensate for increasing shortages of health professionals in some areas. From our point of view, although there is indeed an incentive for the development of telemedicine given the current medical demography in France, when we evaluate the impact of a depressive or psychotic recurrence on the life of a patient, it is clear that technologies that allow better monitoring of their condition should not cause a rejection but rather an interest, especially since these technologies have been developed by doctors (and in a number of cases with patients) and not by politicians. Regarding the first scenario, some respondents argued that this technology could result in the loss of opportunity, especially if it prevented practitioners from diagnosing other problems because the focus was on the system’s diagnosis, and some mentioned the risk of practitioners losing their clinical sensitivity through lack of practice. This type of reasoning applied to other fields of medicine when additional tests or new investigation tools were developed. We do not think that the development of the stethoscope made cardiologists lose their clinical sense, we think that it improved them. These technologies must become complementary psychiatric examinations and complement the currently subjective approach employed by psychiatrists in diagnosis or prediction.

### Assumptions and Recommendations

Significant disparities between devices highlight varying degrees of acceptability, both technology-dependent (previously known technology such as MRI seems better accepted, while connected objects are less well-known) and the underlying theoretical presupposition. Thus, the prediction of a psychotic transition remains subject to many fears, whereas the computerized questionnaires are not. Paradoxically, it is the MRI coupled with ML that psychiatrists find most useful. There are 2 issues very clearly raised here: the psychiatrists questioned in this survey are very minimally informed, notably on the scientificity of these devices, although many studies have already been published and the practitioners do not necessarily know what to do with the new data that these machines can obtain, that is, they have very little knowledge about what to do if there is a risk of psychotic transition, they are not familiar with the concepts of precision medicine, and so on. In this context, it seems very important to increase training measures on new technologies, particularly by integrating this into the resident teaching program. For the past 3 years in France, most psychiatric congresses have offered an innovation session or a new technology session; this seems to be an interesting way to promote these new tools but needs to be expanded.

Furthermore, our results assume that new technologies are challenging the psychological subtype of psychiatrist while consolidating the medical subtype. Neurobiological psychiatrists are not challenged by these technologies, which they regard rather as tools that extend or complement their practice. Regardless of the system, psychiatrists with a psychoanalytic orientation are clearly reluctant, and we suggest that it is both the use of scales and the technological dimension that account for their negative stance. This assumption is reinforced by the large number of comments that evoked the technology’s impact on the therapeutic relationship. Special measures should be taken to reassure that specific subtype of psychiatrist by demonstrating that these systems are part of a global care that does not negate the psychological aspect of the disease and that it allows an improvement in patient care.

Many ethical issues were raised by this study, and data security, data storage, privacy, and hacking risk have yet to be resolved. Disease detection or risk prediction, whether in the case of depressive relapse or transition to psychosis, is necessarily stressful for patients and brings the risk of excessive focus and an anxious counter-reaction. To offset these risks, it is essential for psychiatrists to be involved in the development of new technologies, to prevent their loss of control over them. Developers have a major interest in communicating better about the design of these tools and the algorithms they want to be used in the future. In order to complete our research, a comparative study using the same methodology is being developed to better understand the acceptability of these technologies by patients, nurses, and general practitioners.

### Limitations of the Study

The main limitation could be tautological in that the differences we found between various acceptability profiles may simply have reflected a difference in theoretical approach (neurobiological psychiatrists vs the rest). This suggests that the technologies we studied were based on a theoretical presupposition that was purely neurobiological. While it is true that the third scenario had a clear neurobiological emphasis, the same cannot be said for the other two. In the first scenario, the use of smartphone-based EMA raised the question of scales, but scales presuppose nothing of the etiopathogenesis of the disorder being assessed. Furthermore, CAT does not use scales, as it simulates the psychiatrist’s way of thinking by choosing a specific question from a database made up of more than 500 items. The same applies to the connected wristband as biometric data capture only means that some depression symptoms can be objectified (eg, psychomotor retardation)—a fact that no psychiatrist can refute. It was, therefore, not the opposition between neurobiological and psychodynamic issues that were assessed in our study but the relationship with technology exhibited by different subtypes of psychiatrists.

### Conclusion

The type of professional culture (theoretical background and practice) appeared to exert a strong influence on the acceptability of the technologies we studied. Overall acceptability was moderate, and respondents expressed many reservations and raised many ethical and ideological questions. Indeed, a probability derived from the analyzed data cannot systematically be transformed into diagnostic or therapeutic certainty. Some of their concerns are relevant and their skepticism can be understood although many of the issues raised are in fact devoid of reality and reflect a great lack of knowledge of the current state of research on these new technologies. It is surely necessary for psychiatrists to adopt a clear stance with regard to these radical changes that are upsetting traditional practice, and for them to be able to do this, they must be informed, interested, and allowed to contribute to the development of these new technologies [35,36], going as far as joining the “disruptors of health sciences” [37]. The acceptability model we developed, using a complex sociological methodology featuring clinical case scenarios intended to elicit emotional responses, needs to be replicated.

## Appendix

Multimedia Appendix 1 Screenplay method for the 3 scenarios.

Multimedia Appendix 2 Acceptability model.

Multimedia Appendix 3 Sociodemographic characteristics of respondents and psychiatrists' professional culture.

## Abbreviations

CAT: computerized adaptive testing
CDSS: clinical decision support system
EMA: Ecological Momentary Assessment
ML: machine learning
MRI: magnetic resonance imaging
