# Supplementary material for: Psychiatrists' Attitudes Toward Disruptive New Technologies: Mixed-Methods Study
Source: JMIR Ment Health. 2018 Dec 14;5(4):e10240. doi: 10.2196/10240 (PMC6315247; doi:10.2196/10240)
Supplement: Multimedia Appendix 1 [file mental_v5i4e10240_app1.pdf]

1**Screenplay method**

2**First Scenario (CAT/EMA)**

Mr P, 25 years old, is referred by his general practitioner to Dr D. (liberal psychiatrist) for mood assessment.

On his office, it is proposed to Mr. P. to complete a "CAT-DI" questionnaire on a computer in the waiting room. CAT-DI is an "intelligent" software capable of evaluating depressive symptomatology as accurately as the Hamilton scale (HDRS), but only by asking certain questions and adapting the questions asked to the patient's answers [1]. After analyzing the results of CAT-DI, Dr. D. notes that Mr. P. has a "moderate to severe" depressive symptomatology (se = 0.92 and sp = 0.88).

Dr. D. decided to continue the evaluation to improve the accuracy of his diagnosis. For this, he proposes to download a "PsyEVAL" application on a smartphone, explain how it works and give an appointment the following week to the patient. This application allows an ecological momentary assessment in order to repeatedly evaluate the mood of the patient using PHQ-9 items that appear several times a day on his phone [2].

After 7 days, Mr. P. comes back to Dr. D office and it appears from the data analysis that Mr. P symptomatology is severe and therefore Dr. D. proposes a management adapted to this state.

[1] Gibbons, R. D. et al. Development of a computerized adaptive test for depression. Arch. Gen. Psychiatry 69, 1104-1112 (2012).

[2] Torous, J. et al. Utilizing a Personal Smartphone App to Assess the Patient's Health Questionnaire-9 (PHQ-9) Depressive Symptoms in Patients With Major Depressive Disorder. JMIR Ment Health 2, e8 (2015).

3

4**Second Scenario (Biosensors)**

Mrs. V, 47 years old, has had a recurrent depressive disorder in remission for several months, and she is followed in town once a month by Dr. D (liberal psychiatrist) to renew her treatment. Overburdened by new demands for care, Dr. D. can no longer support this monthly rhythm and he therefore proposes to Mrs. V. to see her again in 3 months.

Anxious not to miss a depressive relapse, he suggested to Mrs. V. to wear a connected watch capable of recording a certain number of biometric data and analyzing their variations (such as a modification of the motor activity, heart rate variability, etc.) which is supposed to detect depression [1] in real time and at a distance, possibly even before the relapse is subjectively detectable [2].

If warning signs are highlighted, the watch sends a signal to Dr. D.'s mobile phone and the doctor can therefore suggest an appointment more quickly to his patient to verify what is happening.

1. Burton, C. et al. Activity monitoring in patients with depression: a systematic review. J Affect Disord 145, 21-28 (2013)

2. https://www.polytechnique.edu/fr/content/une-start-incubee-lx-cree-un-bracelet-pour-detecter-la-depression

5

6 **Third Scenario (MRI/Blood test + ML)**

Mr P., 18 years old, is referred to Dr. D. by his general practitioner because he recently presented an episode described as "psychotic" but spontaneously resolving in 4 days.

Dr. D.'s assessment finds a number of arguments in favor of a BLIP [1] "Brief Limited Intermittent Psychotic Episode", an at-risk mental state for which we know that there is a risk of transition to schizophrenia from 25 to 35% within 3 years [2].

As part of his initial assessment Dr. D. proposes a blood test for the biomarkers of schizophrenia (test with a diagnostic accuracy of 87.9%) [3], and he proposes to complete the assessment a special brain MRI that, coupled with an artificial intelligence, can predict with an accuracy of 84.2% if his patient will go to schizophrenia within 3 years [4].

1. Krebs détection précoce, 2013. Détection précoce des sujets à risque de psychose. http://www.fondation-fondamental.org/upload/pdf/presdetectionprecocecreteilkrebstransmis33.pdf

2. Fusar-Poli, P. et al. The psychosis high-risk state: a comprehensive state-of-the-art review. JAMA Psychiatry 70, 107–120 (2013).

3. Chan, M. K. et al. Development of a blood-based molecular biomarker test for identification of schizophrenia before disease onset. Transl Psychiatry 5, e601 (2015).

4. Koutsouleris, N. et al. Disease prediction in the at-risk mental state for psychosis using neuroanatomical biomarkers: results from the FePsy study. Schizophr Bull 38, 1234–1246 (2012).

7
