# Supplementary material for: Psychiatrists' Attitudes Toward Disruptive New Technologies: Mixed-Methods Study
Source: JMIR Ment Health. 2018 Dec 14;5(4):e10240. doi: 10.2196/10240 (PMC6315247; doi:10.2196/10240)
Supplement: Multimedia Appendix 2 [file mental_v5i4e10240_app2.pdf]

Acceptability model

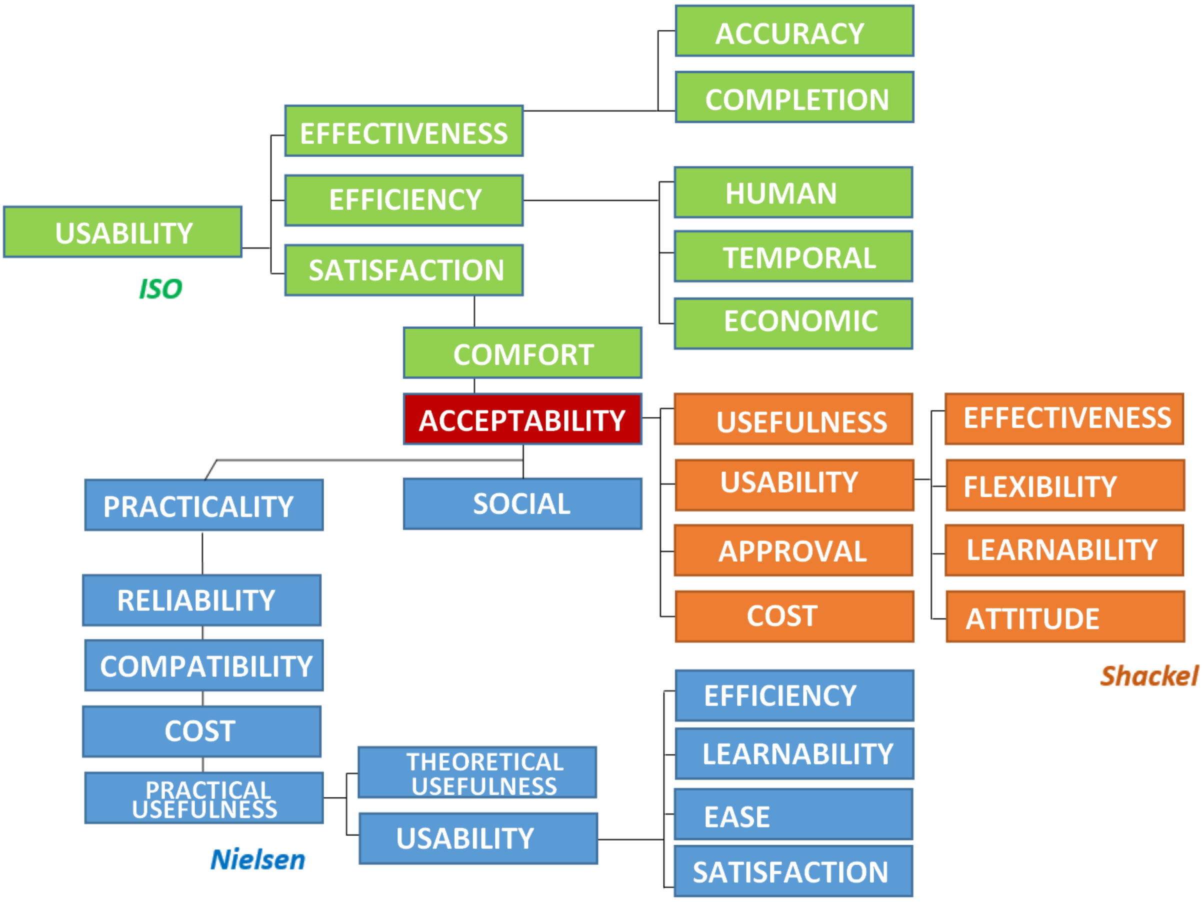

Shackel, B. Human Factors for Informatics Usability. In B. Shackel & B. Richardson (Eds.), Human Factors (pp. 21-38). Cambridge: Cambridge University Press.1991

Nielsen, J. Usability engineering. Boston: Academic Press. 1993

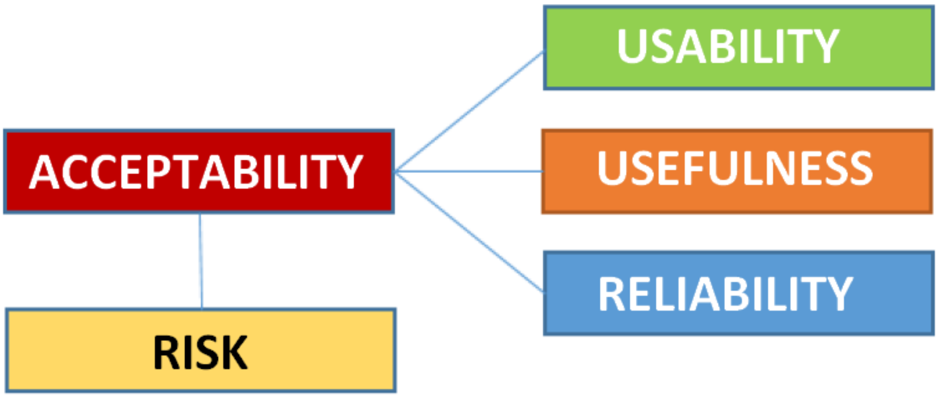

Our model used the primary variable of each model (ISO, Nielsen, Shackel) and add a risk assessment
