# Supplementary material for: Psychiatrists' Attitudes Toward Disruptive New Technologies: Mixed-Methods Study
Source: JMIR Ment Health. 2018 Dec 14;5(4):e10240. doi: 10.2196/10240 (PMC6315247; doi:10.2196/10240)
Supplement: Multimedia Appendix 3 [file mental_v5i4e10240_app3.pdf]

***Characteristics of responders** (N = 515)*	***n*** **(%)**
**Sex**	
Female	299 (58.1)
Male	216 (41.9)
**Year of graduation**	
2016-2020	219 (42.5)
2010-2015	123 (23.9)
2000-2009	63 (12.2)
1990-1999	55 (10.7)
1970-1989	45 (8.7)
Not indicated	10 (1.9)
**Role**	
Resident	241 (46.8)
Hospital practitioner	148 (28.7)
Liberal practitioner	49 (9.5)
Assistant practitioner	41 (8)
Professor	20 (3.9)
Assistant professor	16 (3.1)
**Practice (except residents) [*n* = 274]**	
Psychiatric hospital	99 (36.1)
University hospital	86 (31.4)
Private or clinical practice	38 (13.9)
General hospital	22 (8)
Mixed (private practice and public health service)	15 (5.5)
Other (association, military hospital, etc.)	14 (5.1)
**Theoretical approach**	
Several approaches focusing on neurobiology or CBT	131 (25.4)
Integrative practice (must include neurobiology and psychoanalysis)	114 (22.1)
Neurobiology	74 (14.4)
Psychoanalysis	58 (11.3)
Several approach focusing on psychoanalysis	41 (8)
Cognitive-behavioral therapy (CBT)	41 (8)
Systemic	29 (5.6)
Other	11 (2.1)
Not provided	16 (3.4)
**Area of expertise**	
Adult psychiatry	270 (52.4)
Child psychiatry	91 (17.7)
Mixed (adult and child psychiatry)	51 (9.9)

Addiction medicine	34 (6.6)
Geriatric psychiatry	29 (5.6)
Forensic psychiatry	21 (4.1)
Several areas of practice	17 (3.3)
Not provided	2 (0.4)

<u>Psychiatrists' professional culture</u>

Subtype	Medical	Intermediate	Psychological
**Sex**	Male	*ns*	Female
**Theoretical approach**	Neurobiological	CBT<br>Integrative<br>Systemic	Psychoanalytic
**Practice**	Adult psychiatry<br>Addictology<br>Geriatric psychiatry	*ns*	Child psychiatry<br>Forensic psychiatry
**Role**	Professor<br>Assistant professor	Hospital practitioner<br>Assistant practitioner<br>Resident	Private practitioner
**Year of graduation**	1990-2009	2010-2020	1970-1989
